# A Journalistic Elsler

**Published:** 1886-02

**Authors:** 


A Journalistic Elsler.—The Bible tells us how David of old got himself warmed up; but it took *two* to do it. The modern David of the ancient and venerable *American Practitioner*, having "woed and won a giddy girl," the *Medical News*, has warmed up in a most surprising manner, and the *issue* is a sprightly youth—"just like its pa," but "a little more so;" quite an improvement on the *Am. Prac.* aforesaid. Announcing the coming on of a "performance," which, he says, is to be unusually attractive, (we suggest to call it "May and December"), he says:

"And so the curtain rises on the enlarged and newly decorated stage, while the new combination ["them's um," we suppose] steps up to the footlights, and makes the initial bow."

How realistic! How suggestive of a *ballet* performance! While, in imagination we can almost hear the *cha-cha-cha* of the fiddle, we can see the rejuvinated Miss Fanny, redolent of "bloom of youth," brave in tinsel,and with the merest intimation of skirts, skip on the fantastic with a surprising agility, and without a hint of the *rheumatiz*, turn a *pirouette*, and flash a moment before our raptured and astonished gaze; while we, the journalistic bald-heads, occupying the *front seats*, exclaim, "*bwavo*," oh, "*bwavo*!"

